# Unc93 homolog B1 restricts systemic lethal inflammation by orchestrating TLR7 and TLR9 response

**DOI:** 10.1186/ar3619

**Published:** 2012-02-09

**Authors:** Ryutaro Fukui, Shin-Ichiroh Saitoh, Atsuo Kanno, Masahiro Onji, Takuma Shibata, Akihiko Ito, Morikazu Onji, Mitsuru Matsumoto, Shizuo Akira, Nobuaki Yoshida, Kensuke Miyake

**Affiliations:** 1 Division of Infectious Genetics, Department of Microbiology and Immunology, The Institute of Medical Science, The University of Tokyo, 4-6-1 Shirokanedai, Minatoku, Tokyo 108-8639, Japan; 2 Laboratory of Innate Immunity, The Institute of Medical Science, The University of Tokyo, 4-6-1 Shirokanedai, Minatoku, Tokyo 108-8639, Japan; 3 Laboratory of Developmental Genetics, Center for Experimental Medicine and Systems Biology, The Institute of Medical Science, The University of Tokyo, 4-6-1 Shirokanedai, Minatoku, Tokyo 108-8639, Japan; 4 Department of Pathology, Faculty of Medicine, Kinki University, Osaka 589-8511, Japan; 5 Department of Gastroenterology and Metabology, Ehime University Graduate School of Medicine, Ehime 791-0295, Japan; 6 Division of Molecular Immunology, Institute for Enzyme Research, University of Tokushima, Tokushima 770-8504, Japan; 7 Laboratory of Host Defense, World Premier International Immunology Frontier Research Center, Osaka 565-0871, Japan; 8 Department of Host Defense, Research Institute for Microbial Diseases, Osaka University, Osaka 565-0871, Japan

## 

Nucleotide sensing-TLRs (Toll-like receptors) recognize pathogen derived-nucleic acids and trigger immune response [1]. Because of the highly conserved structure of nucleic acids, these TLRs have risk to recognize host derived-nucleic acids and induce autoimmune disease, therefore it is important to clarify the mechanisms and control the response.

**Figure 1:**
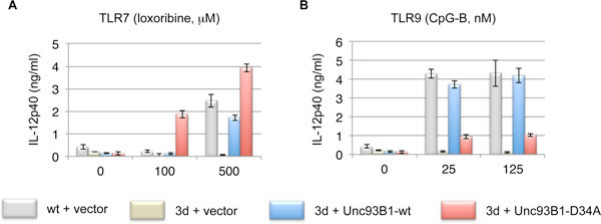
**The D34A mutation of Unc93B1 up-regulates TLR7 response and down-regulates TLR9 response**. **(A and B). **Empty vector was transfected to bone marrow derived stem cells (BMSCs) from wild tipe mice (gray bars). Empty vector (yellow bars), wild type Unc93B1 expressing vector (blue bars), or D34A Unc93B1 expressing vector (red bars) were transfected to BMSCs from 3d mice. Transfected BMSCs were cultured with puromycin and GM-CSF to differentiate to dendritic cells (DCs). After differentiation, DCs were harvested and stimulated by TLR7 ligands (**A**, loxoribine, μg/ml) or TLR9 ligands (**B**, CpG-B, nM). Culture supernatant was corrected and subjected to ELISA for measurement of IL-12p40 (ng/ml).

We found that the responses of TLR7 and TLR9 are balanced reciprocally, and Unc93 homolog B1 (Unc93B1) is a key molecule for this balancing system [2]. Unc93B1 is known as an essential molecule for TLR3, TLR7, and TLR9 responses, and the function depends on its C-terminal region [3]. The balancing function of Unc93B1 is located on 34th aspartic acids from N-terminal, and alanine mutant (D34A) Unc93B1 up-regulates TLR7 response and down-regulates TLR9 response (Figure 1) [2].

It is reported that TLR7 or TLR9 response contributes to some kinds of autoimmune disease and TLR7 overexpressed mice develop SLE like autoimmune disease [4-8]. To investigate the significance of reciprocal TLR7/TLR9 balance in vivo, we generated *Unc93b1*^D34A/D34A ^mice and observed the phenotypes.

**Figure 2:**
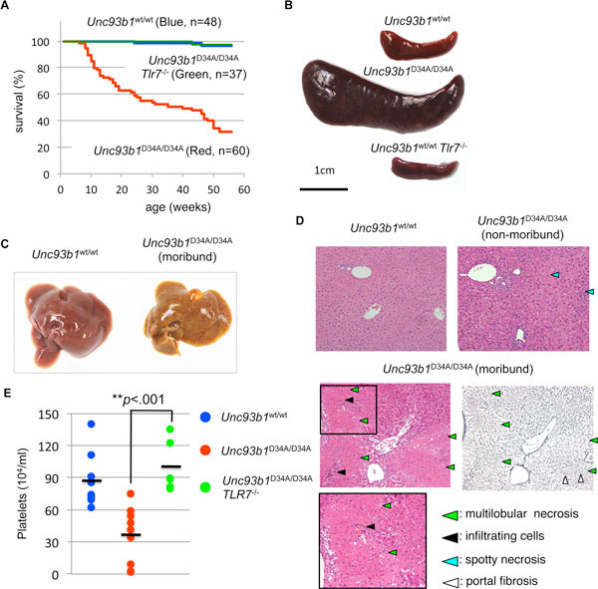
***Unc93b1*^D34A/D34A ^mice develop systemic lethal inflammation spontaneously**. **(A) **Survival curves of *Unc93b1*^WT/WT^, *Unc93b1*^D34A/D34A^, or *Unc93b1*^D34A/D34A^*Tlr7*^-/- ^mice (blue, red, or green line, respectively). **(B and C) **Macroscopic images of spleen **(B) **and liver **(C)**. **(D) **Microscopic analyses of liver. Histological samples were stained by Hematoxyline and Eosine (H&E) or silver impregnation. **(E) **Platelet counts in peripheral blood from indicated genotypes of mice. Bars in the graph indicate averages.

As results, *Unc93b1*^D34A/D34A ^mice were born according to Mendelian rule but started to die spontaneously at 10 weeks old and over half of *Unc93b1*^D34A/D34A ^mice died within 1 year (Figure 2A) [9]. *Unc93b1*^D34A/D34A ^mice developed various phenotypes, for example, splenomegaly, hepatitis, glomerulonephritis, thrombocytopenia, myeloproliferative disorder (Figure 2B-2E). Especially, lethal acute hepatitis was observed in moribund mice and infiltrated myeloid cells in liver were expanded in spleen. These phenotypes are vanished by TLR7 deficient Unc93B1^D34A/D34A ^mice, thus TLR7 hyper-response caused by TLR7/TLR9 balance disruption is factor of phenotypes in *Unc93b1*^D34A/D34A ^mice (Figure 2).

**Figure 3:**
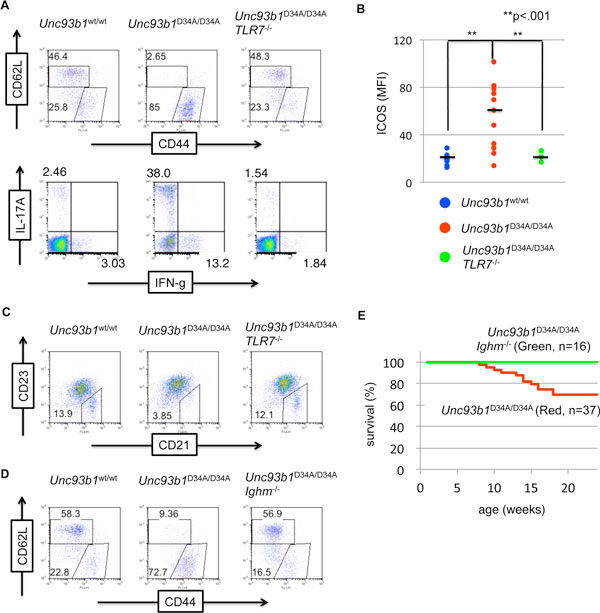
**T cells and B cells are activated in *Unc93b1*^D34A/D34A ^mice**. **(A or D) **Flow cytometry analysis for memory T cells (**A **upper, or **D**) or Th1/Th17 cells (**A **lower). **(B) **Expression of ICOS was measured by cell surface staining of CD4+ T cells. Mean fluorescent intensity (MFI) was calculated and indicated by dots. Black bars in the graph indicate average of MFI. **(C) **Flow cytometry analysis for matrginal zone B cells. **(E) **Survival curves of *Unc93b1*^D34A/D34A ^(Red) or *Unc93b1*^D34A/D34A^*Ighm*^-/- ^(Green) mice.

Not only innate immune system, acquired immune system is also affected by D34A mutation. Expanded memory T cells, up-regulation of ICOS and CD69 on T cells were observed by TLR7 dependent manner and some classes of serum immunoglobulin level is increased in *Unc93b1*^D34A/D34A ^mice. In addition, Th1 and Th17 cells were expanded and activated in *Unc93b1*^D34A/D34A ^mice. The activation of T cells were TLR7 dependent, and mature B cell depleted *Ighm*^-/-^*Unc93b1*^D34A/D34A ^mice did not induce T cell activation and moderated phenotypes (Figure 3D and 3E). It suggests that B cells are activated by TLR7 hyper-response, and the B cells activate T cells to generate phenotypes of *Unc93b1*^D34A/D34A ^mice.However, thrombocytopenia was not completely recovered in *Ighm*^-/-^*Unc93b1*^D34A/D34A ^mice but completely recovered in *Rag2*^-/-^*Unc93b1*^D34A/D34A ^mice. Interaction between cell types and phenotypes should be confirmed as a future plan.
